# Association between dopaminergic polymorphisms and borderline personality traits among at-risk young adults and psychiatric inpatients

**DOI:** 10.1186/1744-9081-6-4

**Published:** 2010-01-12

**Authors:** Zsofia Nemoda, Karlen Lyons-Ruth, Anna Szekely, Eszter Bertha, Gabor Faludi, Maria Sasvari-Szekely

**Affiliations:** 1 Institute of Medical Chemistry, Molecular Biology and Pathobiochemistry, Semmelweis University, Budapest, Hungary; 2 Department of Psychiatry, Harvard Medical School, Cambridge Hospital, Cambridge, Massachusetts, USA; 3 Institute of Psychology, Eotvos Lorand University, Budapest, Hungary; 4 Department of Psychiatry, Kutvolgyi Clinical Centre, Semmelweis University, Budapest, Hungary

## Abstract

**Background:**

In the development of borderline personality disorder (BPD) both genetic and environmental factors have important roles. The characteristic affective disturbance and impulsive aggression are linked to imbalances in the central serotonin system, and most of the genetic association studies focused on serotonergic candidate genes. However, the efficacy of dopamine D2 receptor (DRD2) blocking antipsychotic drugs in BPD treatment also suggests involvement of the dopamine system in the neurobiology of BPD.

**Methods:**

In the present study we tested the dopamine dysfunction hypothesis of impulsive self- and other-damaging behaviors: borderline and antisocial traits were assessed by Structured Clinical Interview for Diagnosis (SCID) for DSM-IV in a community-based US sample of 99 young adults from low-to-moderate income families. For the BPD trait analyses a second, independent group was used consisting of 136 Hungarian patients with bipolar or major depressive disorder filling out self-report SCID-II Screen questionnaire. In the genetic association analyses the previously indicated polymorphisms of the catechol-O-methyl-transferase (COMT Val158Met) and dopamine transporter (DAT1 40 bp VNTR) were studied. In addition, candidate polymorphisms of the DRD2 and DRD4 dopamine receptor genes were selected from the impulsive behavior literature.

**Results:**

The DRD2 TaqI B1-allele and A1-allele were associated with borderline traits in the young adult sample (p = 0.001, and p = 0.005, respectively). Also, the DRD4 -616 CC genotype appeared as a risk factor (p = 0.02). With severity of abuse accounted for in the model, genetic effects of the DRD2 and DRD4 polymorphisms were still significant (DRD2 TaqIB: p = 0.001, DRD2 TaqIA: p = 0.008, DRD4 -616 C/G: p = 0.002). Only the DRD4 promoter finding was replicated in the independent sample of psychiatric inpatients (p = 0.007). No association was found with the COMT and DAT1 polymorphisms.

**Conclusions:**

Our results of the two independent samples suggest a possible involvement of the DRD4 -616 C/G promoter variant in the development of BPD traits. In addition, an association of the DRD2 genetic polymorphisms with impulsive self-damaging behaviors was also demonstrated.

## Background

Borderline personality disorder (BPD) is a severe mental disorder with a high mortality rate as a result of suicide and impulsive behavior [1]. The core symptoms include emotional disturbance (affective instability, intense anger, chronic feelings of emptiness), disturbed cognition (identity disturbance, transient paranoid ideation or dissociative symptoms), impulsivity (suicidal or self-mutilating behavior, or other self-damaging behaviors, such as substance abuse, reckless driving, unsafe sex, spending sprees, binge eating), and interpersonal problems (intense unstable relationships, fear of abandonment). Interaction of multiple genetic factors and distressing childhood experiences has been suggested in the development of emotional dysregulation and impulsivity, leading to BPD symptoms [1]. The first family studies indicated substantial genetic underpinnings [2], and twin studies showed 42-69% heritability estimates [3,4]. Animal and human studies have indicated that reduced serotonin function and increased norepinephrine activity in the brain lead to aggressive and impulsive behavior [5]. Although several impulsive behaviors (e.g. ADHD: Attention Deficit Hyperactivity Disorder or substance abuse) have been linked to altered dopamine function, involvement of the dopamine system in BPD has been circumstantial. The main supporting evidence, thus far, comes from the therapeutic effect of D2 receptor blocking antipsychotic drugs [6].

Hitherto, only a couple of workgroups have studied dopamine system related polymorphisms in BPD. The first genetic association study showing a dopaminergic effect on BPD was conducted among depressed patients [7]. The 9-repeat allele of the dopamine transporter gene (DAT1, SLC6A3) showed significant association with BPD, even when childhood abuse and neglect, and borderline temperament were included in the analyses. A recent study reported an over-representation of the low activity Met/Met genotype of the catechol-*O*-methyl-transferase (COMT) gene in 161 BPD patients [8]. Since COMT metabolizes catecholamines, these findings suggest that altered dopamine and/or norepinephrine neurotransmission might be a contributing factor in the development of BPD. In spite of the pharmacological evidence, dopamine D2 receptor (DRD2) polymorphisms have not been studied in relation to BPD. Dopamine D4 receptor (DRD4) might be another candidate, because of the preferential expression of this D2-family member in the prefrontal cortex [9] and its well-established role in ADHD [10] as well its possible involvement in human personality traits of novelty-seeking and impulsivity [11]. However, a pilot study of 39 BPD patients did not show any significant effect of DRD4 polymorphisms [12].

We aimed to investigate the possible involvement of dopaminergic polymorphisms in the etiology of impulsive self- and other-damaging behaviors (assessed by borderline and antisocial traits) in a previously studied US community-based sample of 99 young adults from low-to-moderate income families [13]. This group could be regarded as an at-risk population, because it has been shown that low family socioeconomic status confers a risk for BPD [14] and low educational or occupational status is an important risk factor for suicide attempts [15]. In this sample 4% were diagnosed with BPD and 7% with antisocial personality disorder (APD) in young adulthood, showing a prevalence 2-3 times higher compared to the general community (BPD: 1-2%, APD: 2-3%) [16,17]. A group of 136 Hungarian patients with mood disorder served as the replication sample for findings related to borderline traits, because borderline diagnosis, as well as borderline traits, have a high prevalence rate among psychiatric inpatients (15%) [16].

In addition to the previously indicated dopaminergic polymorphisms, namely the 40 bp VNTR (variable number of tandem repeats) of the DAT1 gene, and the Val158Met polymorphism of the COMT gene, the most commonly investigated DRD2 and DRD4 polymorphisms were genotyped. The DRD2 TaqIA polymorphism was identified during the chromosomal localization of the gene. However, this single nucleotide polymorphism (SNP) is in the 3' untranslated region, 10 kilobase downstream from the DRD2 gene, actually in the neighboring ANKK1 gene [18]. Nevertheless, four independent workgroups showed reduced D2 receptor density of the minor A1-allele carriers [19-22], and only one reported a negative finding [23]. Therefore, this polymorphism might be a good marker for DRD2 density in the brain. The DRD2 gene has been associated with alcoholism and other addictive disorders [24], and is hypothesized to be a reinforcement or reward gene. Recently, two studies showed an association between the DRD2/ANKK1 A1-allele and impulsive behavior among healthy young adults [25,26]. To further assess the possible involvement of the DRD2 gene, two additional SNPs (TaqIB from intron 1 and TaqID from intron 2) were selected for genotyping.

Within the DRD4 gene, the 48 bp VNTR in exon III has received the most interest in psychiatric genetics [10,11]. Recent studies suggested that the 7-repeat allele of the DRD4 gene may result in reduced DRD4 expression, influencing RNA stability [27]. Therefore, other polymorphisms located in the promoter region of the DRD4 gene with predictable or proven effects on gene expression might also be worthwhile to study. The -616 C/G and -521 C/T SNPs, and a 120 bp duplication in the 5' untranslated region of the DRD4 gene have been extensively studied in dopamine-related child psychiatric disorders, such as ADHD [10]. Therefore, we have analyzed these four DRD4 gene variants in the two samples.

Since gene × gene and gene × environment interactions may be important in the development of psychiatric disorders, exploratory analyses investigating previously reported interactions in the impulsive behavior literature were also conducted. For example, DRD2 A1-allele × DRD4 7-repeat allele interaction has been reported for impulsivity [25], sensation seeking [28], and antisocial behavior [29], and DRD4 VNTR × DAT1 VNTR interaction has been reported for behavioral inhibition [30]. Also, environmental risk and protective factors, such as maternal insensitivity or intervention promoting positive parenting, were shown to interact with the DRD4 7-repeat allele on child externalizing (oppositional, aggressive) behavior [31,32].

## Methods

### US sample of young adults

In the US sample 99 young adults from low-to-moderate income families (59.6% female; age range 18-22) participated in a study of adolescent-parent relationships [13]. Forty one of the families had been participating in a longitudinal study of attachment relationships since infancy, and were recruited at child age 0-18 months. All 41 families were at or below federal poverty level at intake, and half of the families were referred to clinical services during the young adult's infancy for concerns about the quality of care provided (the other half were socioeconomically-matched controls). An additional 48 crossectional families were recruited as part of the young adult follow-up study and matched to the longitudinal sample on socioeconomic status. Hence, the combined study sample of 99 young adults is regarded as an at-risk sample (53.5% of the families had an income of $40,000 per year or less; 43.4% of mothers had a high school education or less; 37.4% were single parents). The study was conducted in compliance with the Code of Ethics of the World Medical Association and with the requirements of the Hospital Institutional Review Board and the National Institute of Mental Health. All young adults and their mothers provided written informed consent for their participation.

Ethnicity/race data was obtained from the mother of the young adult during the interview by asking her to classify the ethnicity and race of both herself and the father (possible options for ethnicity: Hispanic or Latino; Not Hispanic or Latino; possible alternatives for race: American Indian or Alaska Native; Black or African American; White; Asian; Native Hawaiian or other Pacific Islander; more than one race). Of the 99 young adults, 66 had two parents reported as White, 26 subjects had one or two African American parents, and 7 subjects had one or two parents who were Hispanic. The genetic analyses yielding significant association for the total sample (N = 99) were repeated for the non-Hispanic Caucasian subgroup (N = 66).

### US sample symptom assessments

Young adult psychopathology was assessed using the Structured Clinical Interview for Diagnosis (SCID) for DSM-IV Axis I and II [33,34], which was administered in the laboratory by trained clinicians (see clinical characteristics for Axis I disorders in Table 1). Among the 99 young adults 2 were diagnosed with both BPD and APD, 2 with BPD, 5 with APD, and 14 with other personality disorder(s). The pattern of antisocial or borderline personality disorder traits among the young adults were the following: 4% met criteria for a diagnosis of BPD, however, 20% endorsed two or more borderline symptoms; 7% of the sample met diagnosis of APD (i.e. 3 or more APD symptoms and 2 or more conduct disorder symptoms before age 15 years), and 26% showed two or more antisocial symptoms. In the categorical analyses (chi-square and binary logistic regression) borderline and antisocial traits were coded as present if two or more features of the disorder were endorsed.

**Table 1:** Clinical characteristics in the US young adult sample and in the Hungarian sample of depressive patients

DSM-IV Axis I diagnoses	US sample N (%)	Hu sample N (%)
Major Depression*	23 (23.2)	79 (58.1)
Bipolar Disorder	7 (7.1)	50 (36.8)
Schizophrenia	0 (0)	4 (2.9)
Schizoaffective Disorder	0 (0)	3 (2.2)
Anxiety Disorder	40 (40.4)	85 (62.5)
Substance Abuse	38 (38.4)	12 (8.8)
Psychotic symptoms	24 (24.2)	76 (55.9)

* including single episode

In the analyses a severity of abuse variable was also used: An overall rating for severity of abuse from birth to age 18 was assigned by reviewing the Conflict Tactics Scale second version [35], the Traumatic Stress Schedule [36], and the Childhood Traumatic Experiences Scales-Revised coded from one-hour semi-structured Adult Attachment Interview [37], and any history of state protective services involvement. The severity of abuse scale was coded as 0 - no occurrence of violence; 1 - harsh punishment or witnessed violence; 2 - emotional/verbal abuse; 3 - one type of physical or sexual abuse (using state guidelines for abuse), or protective services/foster care involvement; 4 - two or more forms of abuse/trauma out of the three listed previously. Reliability of the severity of abuse scale was assessed by having two coders independently rate 37 of the 99 participants on severity of abuse by reviewing the above set of assessments; reliability between the two coders assessed by intraclass correlation was high, r_i _= 0.99.

### Hungarian patient sample

The independent replication sample consisted of psychiatric inpatients diagnosed with mood disorders, recruited from the Department of Psychiatry, Kútvölgyi Clinical Centre (Budapest, Hungary) during their depressive periods requiring treatment. Unrelated participants of Caucasian (Hungarian) origin were included in the present study, thus creating an ethnically homogenous population [38]. The study was conducted in compliance with the Helsinki Declaration, and was approved by the Local Ethical Committee (TUKEB), all participants provided written informed consent. After screening out organic causes of psychiatric disorders, DSM-IV psychiatric diagnoses [39] were obtained by a clinical psychiatrist, the Axis I disorder frequencies are presented in Table 1. Among the 136 patients (female 76.5%; mean age: 46.3 ± 10.5, age range: 21-64 years) 14 (10.3%) were diagnosed with personality disorders. For the genetic analyses borderline symptoms were assessed from the self-report SCID Screen questionnaire, which showed good correlations with SCID-II interviews [40]. The number of BPD symptoms (0-9) was used as the borderline scale; the internal consistency of the scale was found to be satisfactory (Cronbach's alpha: 0.729).

### Genotyping and genotype grouping

Non-invasive DNA sampling was applied, and DNA was isolated from buccal cells using the DNA-purification kit obtained from Gentra (Minneapolis, US). Genotyping procedures for the DRD2 TaqIA (rs1800497), TaqIB (rs1079597), TaqID (rs1800498), and DAT1 40 bp VNTR in the 3' untranslated region were carried out using published protocols [41-44]. Whereas VNTR genotyping methods of the DRD4 48 bp VNTR in exon III and 120 bp duplication in the promoter region, and the allele-specific amplifications of the DRD4 -616 C/G (rs747302), -521 C/T (rs1800955) and COMT Val158Met (rs4680) were developed and optimized in our laboratory [45-47]. The genotyping accuracy was checked by parallel genotyping of two independent DNA samples per person, and by calculating chi-square tests for deviation from the Hardy-Weinberg equilibrium using Knud Christensen's program [48]. Except for the -616 C/G SNP in the Hungarian psychiatric sample, no significant deviations from the Hardy-Weinberg equilibrium were detected in either populations. We assume that the slight deviation from the equilibrium (χ^2 ^= 4.099, df = 1, p = 0.043) in the Hungarian patient sample originates from possible association of the -616 C/G SNP with psychiatric disorder(s). In order to support this assumption, control subjects (with no psychiatric history) were recruited at the Institute of Psychology, Eotvos Lorand University (N = 178, age: 23.2 ± 5.0; 72.5% female) and genotyped for the studied dopaminergic polymorphism (see additional file 1). Our data demonstrated that the non-clinical Hungarian sample was indeed in Hardy-Weinberg equilibrium for -616 C/G SNP (χ^2 ^= 0.104, df = 1, p = 0.747).

In the genetic analyses the VNTRs were analyzed according to the number of the risk allele, i.e. the DRD4 48 bp VNTR 7-repeat allele and the DAT1 40 bp VNTR 9-repeat allele (see Table 2 and 3). In the second set of analyses the genotypes were grouped according to the presence of the risk allele, resulting in DRD4 7-present (7+) and 7-absent (7-) groups. At the DAT1 VNTR the grouping was based on the presence of the 9-repeat allele, resulting in 9-present (9+) and 9-absent (9-) groups; the rare 3/10, 6/10, 10/11, and 10/12 genotype were grouped together with the 10/10 genotype in the 9- group, whereas the rare 3/9 and 8/9 genotype were regarded as having one 9-repeat allele, and grouped to the 9+ group. At the SNP markers, homozygotes for the minor allele were grouped together with heterozygotes in the second round of analyses, i.e. DRD2 A1+ group consisted of A1/A1 (TT) and A1/A2 (CT) genotypes, DRD2 B+ group consisted of B1/B1 (AA) and B1/B2 (AG) genotypes. The DRD4 -521 C/T SNP was analyzed in CC + CT vs. TT setting, and at the -616 C/G SNP CC vs. CG + GG groups were compared.

**Table 2:** Genetic analyses of the dopaminergic polymorphisms in the US young adult sample

		BPD trait (N = 99)					APD trait (N = 98)				
		
		0 or 1		2 or more			0 or 1		2 or more		
		**N**	**(%)**	**N**	**(%)**	**p**	**N**	**(%)**	**N**	**(%)**	**p**

**COMT**	Met/Met	12	(15.2)	4	(20.0)		12	(16.7)	4	(15.4)	
	Met/Val	38	(48.1)	7	(35.0)	0.572	30	(41.7)	14	(53.8)	0.539
	Val/Val	29	(36.7)	9	(45.0)		30	(41.7)	8	(30.8)	

**DAT1**	9/9	9	(11.5)	1	(5.0)		8	(11.3)	2	(7.7)	
40 bp	one 9	25	(32.1)	7	(35.0)	0.689	25	(35.2)	7	(26.9)	0.576
VNTR	no 9	44	(56.4)	12	(60.0)		38	(53.5)	17	(65.4)	

**DRD2**	B1/B1	0	(0.0)	1	(5.0)		0	(0.0)	1	(3.8)	
TaqIB	B1/B2	15	(19.5)	11	(55.0)	**0.001**	17	(24.3)	9	(34.6)	0.137
	B2/B2	62	(80.5)	8	(40.0)		53	(75.7)	16	(61.5)	
	
	B1+	15	(19.5)	12	(60.0)		17	(24.3)	10	(38.5)	
	B1-	62	(80.5)	8	(40.0)	**0.0003**	53	(75.7)	16	(61.5)	0.170

TaqID	C/C	22	(28.6)	5	(25.0)		21	(30.0)	6	(23.1)	
	C/T	32	(41.6)	12	(60.0)	0.276	27	(38.6)	17	(65.4)	**0.046**
	T/T	23	(29.9)	3	(15.0)		22	(31.4)	3	(11.5)	

TaqIA	A1/A1	3	(3.8)	3	(15.0)		4	(5.6)	2	(7.7)	
	A1/A2	26	(32.9)	12	(60.0)	**0.005**	25	(34.7)	13	(50.0)	
	A2/A2	50	(63.3)	5	(25.0)		43	(59.7)	11	(42.3)	0.310
	
	A1+	29	(36.7)	15	(75.0)		29	(40.3)	15	(57.7)	
	A1-	50	(63.3)	5	(25.0)	**0.002**	43	(59.7)	11	(42.3)	0.126

**DRD4**	1/1	4	(5.1)	1	(5.0)		3	(4.2)	2	(7.7)	
120 bp	1/2	26	(32.9)	9	(45.0)	0.592	26	(36.1)	9	(34.6)	0.782
dup	2/2	49	(62.0)	10	(50.0)		43	(59.7)	15	(57.7)	

-616	C/C	14	(17.7)	9	(45.0)		14	(19.4)	9	(34.6)	
	C/G	41	(51.9)	9	(45.0)	**0.020**	39	(54.2)	11	(42.3)	0.289
	G/G	24	(30.4)	2	(10.0)		19	(26.4)	6	(23.1)	
	
	C/C	14	(17.7)	9	(45.0)		14	(19.4)	9	(34.6)	
	C/G + G/G	65	(82.3)	11	(55.0)	**0.010**	58	(80.6)	17	(65.4)	0.118

-521	C/C	10	(12.7)	3	(15.0)		11	(15.3)	2	(7.7)	
	C/T	46	(58.2)	6	(30.0)	0.061	42	(58.3)	10	(38.5)	**0.038**
	T/T	23	(29.1)	11	(55.0)		19	(26.4)	14	(53.8)	
	
	C/C + C/T	56	(70.9)	9	(45.0)		53	(73.6)	12	(46.2)	
	T/T	23	(29.1)	11	(55.0)	**0.029**	19	(26.4)	14	(53.8)	**0.011**

48 bp	7/7	2	(2.5)	2	(10.0)		3	(4.2)	1	(3.8)	
VNTR	one 7	20	(25.3)	5	(25.0)	0.314	17	(23.6)	8	(30.8)	0.773
	no 7	57	(72.2)	13	(65.0)		52	(72.2)	17	(65.4)	

The at-risk population of young adults was divided by the cut-off rate of 2 symptoms of borderline personality disorder (BPD) and 2 symptoms of antisocial personality disorder (APD). For the VNTRs, genotype grouping was based on the number of DAT1 40 bp VNTR 9-repeat alleles and DRD4 48 bp VNTR 7-repeat alleles. For the DAT1 VNTR, one DNA sample, for the DRD2 TaqIB and TaqID SNPs, two DNA samples did not give genotype result (total sample size therefore was 98 or 97 in BPD and 97 or 96 in APD, respectively).

**Table 3:** Dopaminergic polymorphisms and borderline personality symptoms in the Hungarian patient sample

	Genotype	N	BPD score ± STD	F	df	p	η^2^	power
**COMT**	Met/Met	34	4.79 ± 2.54	0.367	2,131	0.694	0.006	0.108
	Met/Val	70	4.71 ± 2.29					
	Val/Val	32	5.16 ± 2.57					

**DAT1**	9/9	11	4.64 ± 2.87	0.120	2,131	0.887	0.002	0.068
40 bp	9/10	48	4.75 ± 2.74	0.082	1,132	0.775	0.001	0.059
VNTR	10/10*	77	4.92 ± 2.13					

**DRD2**	B1/B1	3	3.33 ± 3.22	1.712	2,131	0.184	0.025	0.355
TaqIB	B1/B2	37	5.38 ± 2.31	1.518	1,132	0.220	0.011	0.231
	B2/B2	96	4.68 ± 2.40					

TaqID	C/C	27	4.30 ± 2.54	1.490	2,131	0.229	0.022	0.313
	C/T	59	5.31 ± 2.28					
	T/T	50	4.58 ± 2.43					

TaqIA	A1/A1	4	3.75 ± 2.75	0.460	2,131	0.632	0.007	0.124
	A1/A2	41	4.90 ± 2.56	0.001	1,132	0.970	0	0.050
	A2/A2	91	4.86 ± 2.34					

**DRD4**	1/1	4	3.25 ± 2.06	1.611	2,131	0.204	0.024	0.336
120 bp	1/2	40	5.18 ± 2.18	1.604	1,132	0.208	0.012	0.242
dup	2/2	92	4.76 ± 2.50					

-616	C/C	44	5.48 ± 2.45	5.146	2,131	**0.007**	0.073	0.818
	C/G	56	4.80 ± 2.36	8.152	1,132	**0.005**	0.058	0.809
	G/G	36	4.11 ± 2.27					

-521	C/C	34	5.00 ± 2.54	0.463	2,131	0.630	0.007	0.124
	C/T	65	4.62 ± 2.38	0.354	1,132	0.553	0.003	0.091
	T/T	37	5.08 ± 2.37					

48 bp	7/7	3	5.67 ± 1.53	0.282	2,131	0.754	0.004	0.094
VNTR	one 7	35	4.77 ± 2.38	0.134	1,132	0.715	0.001	0.065
	no 7	98	4.84 ± 2.45					

Haplotype	0	68	4.40 ± 2.34	3.602	2,131	**0.030**	0.052	0.658
-616 C ~	1	50	5.14 ± 2.46	6.969	1,132	**0.009**	0.050	0.746
-521 T	2	18	5.67 ± 2.30					

The mean ± SD of the sum of borderline symptoms (BPD score) from the self-report SCID-II Screen questionnaire are presented for the different genotype groups. Where it is indicated, in the second set of univariate ANOVAs rare homozygote genotypes (n < 5) or literature-based genotypes were grouped together with the heterozygotes for the fixed factor; sex and age were used as covariates.

* At the DAT1 VNTR one 10/11 and one 10/12 sample were grouped together with the 10/10 (9-allele absent) genotype group.

### Statistical analyses

The SPSS program for Windows (17.0 version) was used for the statistical analyses. In the US young adult sample Pearson chi-square analyses were conducted using the symptom cut-off scores at 2 to assess the effect of dopaminergic polymorphisms. In the binary logistic regression analyses gender and severity of abuse were also entered beside the dichotomous genetic variable in the same step (block 1). In the exploratory interaction analyses of DRD4 48 bp VNTR, dichotomous genetic variables (DRD4 7+ vs 7-, DRD2 A1+ vs A1-, DAT1 9+ vs 9-), gender, and the 5-point scale of severity of abuse were entered at block 1, whereas the created interaction variable (DRD4 × DRD2, DRD4 × DAT1, or DRD4 × abuse) was entered in block 2. Genetic association analyses within the Hungarian clinical sample were conducted by univariate analysis of variance using the borderline scale as the dependent variable and the dopaminergic genotypes as the grouping factor, with sex and age used as covariates. To control for multiple comparisons, the False Discovery Rate adjustment of significance levels was used at α = 0.05 level [49].

Haplotype analyses were conducted by likelihood-based association analysis using the Unphased program [50], also direct molecular haplotyping was applied for the DRD4 promoter -616 C/G and -521 C/T SNPs [46]. Linkage disequilibrium between the DRD2 SNPs was calculated by the Haploview program [51] using the Hungarian control sample genotype data: the DRD2 TaqIB SNP was in linkage with TaqIA (D'= 0.947, LOD = 29.77, r^2 ^= 0.701) and with TaqID (D'= 1.0, LOD = 12.17, r^2 ^= 0.212). Linkage disequilibrium for the DRD4 promoter polymorphisms has been previously reported [46].

## Results

### Association analyses among at-risk US young adults

The previously indicated polymorphisms in the DAT1 and COMT gene were not associated with either borderline or antisocial traits in the US young adult sample (Table 2). However, both the DRD2 B1-allele and A1-allele were significantly associated with borderline traits (TaqIB 3 genotypes: χ^2 ^= 14.935, df = 2, p = 0.001, B1+ vs B1- categories: χ^2 ^= 12.977, df = 1, p = 0.0003; TaqIA 3 genotypes: χ^2 ^= 10.568, df = 2, p = 0.005, A1+ vs A1- categories: χ^2 ^= 9.477, df = 1, p = 0.002). These associations remained significant after correcting for multiple comparisons (p < 0.0056). Subjects carrying at least one B1-allele were six times more likely to develop borderline symptoms, OR = 6.2 (2.15-17.85), whereas A1+ subjects were five times more likely to have borderline symptoms, OR = 5.17 (1.7-15.7). Similar results were found in the Caucasian subgroup (N = 66): TaqIB 3 genotypes: χ^2 ^= 15.415, df = 2, p = 0.0004; B1+ vs B1- categories: χ^2 ^= 14.139, df = 1, p = 0.0002, OR = 10.1 (2.7-37.6). TaqIA 3 genotypes: χ^2 ^= 11.253, df = 2, p = 0.004; A1+ vs A1- categories: χ^2 ^= 11.215, df = 1, p = 0.001, OR = 7.7 (2.13-27.99). With gender and severity of abuse accounted for in the model, the effect of DRD2 SNPs was still significant (Table 4). Similar results appeared in the Caucasian subgroup (Table 4). For the APD traits, only the DRD2 TaqID SNP showed a nominally significant association (p = 0.046) which did not remain significant after correcting for multiple comparisons.

**Table 4:** Logistic regression analyses in the US young adult sample

	Total sample (N = 99)			Caucasians (N = 66)		
	
	OR	(95% CI)	p	OR	(95% CI)	p
**DRD2 TaqIB**						

gender	1.91	(0.61 - 5.97)	0.268	1.96	(0.47 - 8.20)	0.358

abuse	1.74	(1.15 - 2.61)	0.008	1.76	(1.08 - 2.89)	0.025

**3 genetic groups**	**6.80**	**(2.14 - 21.57)**	**0.001**	**10.84**	**(2.56 - 45.90)**	**0.001**

gender	1.97	(0.63 - 6.15)	0.241	2.05	(0.50 - 8.51)	0.322

abuse	1.76	(1.17 - 2.65)	0.006	1.80	(1.10 - 2.93)	0.018

**2 genetic groups**	**7.00**	**(2.18 - 22.46)**	**0.001**	**11.20**	**(2.62 - 47.86)**	**0.001**

**DRD2/ANKK1 TaqIA**						

Gender	2.25	(0.75 - 6.76)	0.148	2.39	(0.62 - 9.22)	0.207

Abuse	1.59	(1.08 - 2.34)	0.019	1.59	(1.01 - 2.52)	0.048

**3 genetic groups**	**3.27**	**(1.36 - 7.83)**	**0.008**	**3.70**	**(1.28 - 10.69)**	**0.016**

Gender	2.25	(0.75 - 6.74)	0.147	2.71	(0.69 - 10.66)	0.154

Abuse	1.61	(1.10 - 2.37)	0.015	1.67	(1.05 - 2.65)	0.031

**2 genetic groups**	**4.91**	**(1.54 - 15.64)**	**0.007**	**7.44**	**(1.91 - 28.98)**	**0.004**

**DRD4 -616 C/G**						

Gender	1.90	(0.60 - 6.01)	0.274	1.92	(0.46 - 8.00)	0.371

Abuse	2.12	(1.36 - 3.31)	0.001	2.16	(1.28 - 3.65)	0.004

**3 genetic groups**	**4.64**	**(1.75 - 12.30)**	**0.002**	**5.40**	**(1.66 - 17.57)**	**0.005**

Gender	1.64	(0.53 - 5.13)	0.392	1.85	(0.46 - 7.53)	0.390

abuse	2.06	(1.31 - 3.22)	0.002	2.04	(1.23 - 3.40)	0.006

**2 genetic groups**	**7.65**	**(1.92 - 30.53)**	**0.004**	**7.98**	**(1.69 - 37.72)**	**0.009**

**DRD4 -521 C/T**						

gender	2.08	(0.70 - 6.12)	0.185	2.70	(0.73 - 9.95)	0.137

abuse	1.66	(1.14 - 2.43)	0.008	1.76	(1.12 - 2.78)	0.015

**3 genetic groups**	**1.50**	**(0.63 - 3.56)**	**0.359**	**1.89**	**(0.66 - 5.38)**	**0.235**

gender	1.92	(0.64 - 5.71)	0.244	2.82	(0.73 - 10.87)	0.131

abuse	1.70	(1.15 - 2.51)	0.008	1.85	(1.14 - 3.00)	0.013

**2 genetic groups**	**2.67**	**(0.90 - 7.90)**	**0.077**	**3.50**	**(0.96 - 12.72)**	**0.057**

Among the DRD4 polymorphisms, the -616 C/G and -521 C/T promoter SNPs showed potentially interesting associations with borderline and antisocial traits (Table 2). The -616 CC and -521 TT genotypes were overrepresented among those who had 2 or more borderline or antisocial symptoms. In the second round of analyses homozygotes for the minor allele were grouped together with heterozygotes. Using the CC vs CG + GG grouping system at the -616 C/G SNP, the difference in genotype distribution was nominally significant for borderline symptoms (p = 0.010), whereas at the -521 C/T SNP the CC + CT vs TT grouping showed nominally significant results for both borderline (p = 0.029) and antisocial (p = 0.011) symptoms. In the Caucasian subgroup similar finding emerged for the -616 C/G SNP when testing borderline symptoms (χ^2 ^= 6.732, df = 1, p = 0.009). Whereas at the -521 C/T SNP the analyses showed only marginal effect (borderline: χ^2 ^= 3.609, df = 1, p = 0.057, antisocial: χ^2 ^= 3.493, df = 1, p = 0.062). Neither of the nominally significant associations (p < 0.05) remained significant after correcting for multiple comparisons (p < 0.0056). However, in the logistic regression analyses (entering gender and severity of abuse in the model), the -616 C/G SNP had a significant effect on BPD symptoms at p < 0.0056 level (Table 4). Similar results emerged in the Caucasian subgroup: severity of abuse and the number of -616 C allele were significant predictors of BPD symptoms (p = 0.004, and p = 0.005, respectively, Table 4). The DRD4 48 bp VNTR did not show any association with either BPD or APD traits (Table 2).

Since dopaminergic polymorphisms have been associated with alcohol and drug abuse, we checked for possible confounding genetic association with substance abuse in our sample. Among the young adults 38.4% were diagnosed with substance abuse (Table 1). At the DRD2 SNPs the association of the TaqIA polymorphism with substance abuse was marginally significant using the 3 genotype categories (χ^2 ^= 5.34, df = 2, p = 0.069) and nominally significant for the A1+ vs A1- categories (χ^2 ^= 4.52, df = 1, p = 0.034), with 57.9% of the substance abuse present group carrying the A1-allele vs 36.1% of the substance abuse absent group. Similar findings emerged at the TaqIB 3 genotype categories: χ^2 ^= 5.07, df = 2, p = 0.079, and at the B1+ vs B1- categories: χ^2 ^= 4.21, df = 1, p = 0.04, with 39.5% of the substance abuse present group carrying the B1-allele vs 20.3% of the substance abuse absent group. These results did not remain significant after correcting for multiple testing. None of the DRD4 promoter SNPs showed significant association with substance abuse using either the 3 or 2 genotype categories.

### Exploratory interaction analyses

According to previously reported findings, exploratory interaction analyses were conducted with the DRD4 48 bp VNTR (for genotype grouping and analysis description see Methods section). The DRD4 7-repeat allele × severity of abuse interaction analysis did not yield a significant result (p = 0.43). Nor did the DRD4 7+ × DRD2 A1+ interaction analysis result in a significant finding (p = 0.725). There was a nominally significant finding at the DRD4 7+ × DAT1 9+ interaction (p = 0.045), but this result did not remain significant after correcting for multiple testing.

### Association analyses in the Hungarian inpatient psychiatric sample

For confirmation of the findings regarding borderline traits, univariate ANOVA was conducted in an independent Caucasian population, using the SCID-II Screen questionnaire borderline scale in a Hungarian sample of depressive patients (Table 3). Neither of the DRD2 polymorphisms showed association with BPD traits. On the other hand, the DRD4 -616 C/G SNP showed similar association with BPD traits as observed among the at-risk US young adults. The -616 CC genotype was associated with more severe borderline symptoms using the three genotype groups (p = 0.007). Results were also significant for the combined CC vs CG + GG genotype grouping (p = 0.005). Other dopaminergic polymorphisms did not show any significant associations with the number of borderline symptoms in this clinical sample.

### Haplotype analyses

Since both the DRD2 TaqIB and TaqIA SNPs were associated with BPD traits, haplotype analyses were conducted using the Unphased program. Again, only the US young adult sample analysis yielded significant results (likelihood ratio χ^2 ^= 12.49, df = 2, p = 0.002), with the B1 ~ A1 (A ~ T) haplotype conferring risk for BPD traits: OR = 4.89 (2.05 - 11.66). Furthermore, the haplotype constructed from the three DRD2 SNPs was also significantly associated with BPD traits: likelihood ratio χ^2 ^= 13.91, df = 4, p = 0.008, OR = 7.35 (2.38 - 22.7) for the A ~ C ~ T haplotype. The estimated haplotype analyses for the DRD4 -616 C and -521 T alleles showed association with BPD traits in both samples: US young adult sample: likelihood ratio χ^2 ^= 10.41, df = 3, p = 0.015; Hungarian patient sample: likelihood ratio χ^2 ^= 9.81, df = 3, p = 0.02. In addition, the exact chromosomal localization of the two DRD4 SNPs could be determined by direct haplotyping methods [46]. Genetic association analyses were conducted using the number of the -616C ~ -521T (C ~ T) haplotype. In the US young adult sample the frequency of the DRD4 -616C ~ -521T haplotype was increased among those who had 2 or more borderline symptoms (55% had one C ~ T and 25% had two C ~ T haplotype vs 47.4% and 5.1%, respectively, χ^2 ^= 9.89, df = 2, p = 0.007). In the logistic regression analyses, with gender and severity of abuse accounted for in the model, the genetic effect was still significant: OR = 4.76 (1.77 - 12.79), p = 0.002. In the Hungarian patient sample univariate ANOVA showed a similar effect: those who had -616C ~ -521T haplotype on one or two chromosomes displayed a higher number of borderline symptoms (for three groups: p = 0.03, for two groups: p = 0.009, Table 3).

## Discussion

Here we demonstrated a significant association between the DRD4 -616 C/G promoter polymorphism and borderline traits in two independent samples. Low-income young adults as well as psychiatric patients carrying the CC genotype displayed more borderline symptoms. Moreover, the DRD4 promoter SNP combination of -616C ~ -521T showed association with BPD traits in both groups. Two DRD2 SNPs (TaqIA and TaqIB) were also associated with borderline traits among at-risk young adults, and the haplotype analyses confirmed the role of the DRD2 gene. However, neither of the DRD2 polymorphisms had any significant effect in the sample of psychiatric patients.

Since impulsive self-damaging behaviors constitute one of the core features of BPD, our DRD2 findings might support the association between the DRD2/ANKK1 A1-allele and impulsive behavior reported in healthy young adults [25,26]. Checking for the most frequent symptoms in the studied groups indicated that US young adults displaying two or more borderline symptoms were indeed more likely to exhibit the two forms of impulsivity, namely criterion 4 (impulsive self-damaging behaviors, 75%) and criterion 5 (suicidal or self-mutilating behavior, 50%). Another frequent symptom in this group was intense and unstable relationships (55%). Interestingly, the DRD2 genetic association results can also be related to this phenotype, since pair bonding in monogamous rodents is partially linked to D2 receptors in nucleus accumbens and to the mesolimbic dopamine reward system [52]. Moreover, the A1-allele has been associated with pair-bonding behaviors in humans, as individuals with this low-expression DRD2 allele were less likely to want to marry or have children [28]. This observation is in agreement with animal studies where administration of a DRD2 antagonist inhibited pair bond formation [reviewed in [53]].

On the other hand, depressive patients with borderline features displayed more symptoms from the emotional disturbance domain (chronic feelings of emptiness: 72.1%, affective instability: 67.6%, intense anger: 67.6%). Also, paranoid ideation or dissociative symptoms were quite frequent (68.4%) in this group. Based on the differences in BPD symptoms and in the genetic association findings, we hypothesize that the DRD4 genetic association (present in both samples) might indicate a dopaminergic vulnerability to BPD symptom development involving the D4 dopamine receptor expressed preferentially in the prefrontal cortex. Whereas the DRD2 genetic findings (present only among young adults) are related more to the impulsive phenotype and/or to less effective pair bonding, with an altered striatal D2 dopamine receptor neurotransmission in the background. The nominally significant effects of DRD2 polymorphisms on substance abuse in our sample might also relate to the association between DRD2 and impulsive self-damaging behaviors.

Limitations of the present study include the differences in the studied groups (community sample of young adults vs middle-aged inpatients) and the differences in symptom assessments (SCID-II interview vs self-report SCID-II Screen questionnaire). Another limiting factor in the interpretation of our results is the high rate of comorbid conditions, such as substance abuse in the US young adult sample or anxiety disorder in the Hungarian patient sample. Therefore, further analyses are required to assess the genetic effects on specific BPD symptoms in a wide range of subjects, probably involving healthy individuals without confounding comorbid conditions.

We did not detect any significant association between the investigated COMT or DAT1 polymorphisms and borderline symptoms in either the US young adult sample or the Hungarian psychiatric patient population, although the DAT1 9-repeat allele has been linked not only to BPD [7] but also to impulsivity [54]. The latter workgroup also showed higher reward-related ventral striatum reactivity (which was associated with self-reported impulsivity) in subjects with at least one DRD4 7-repeat allele and in DRD2 -141C Del carriers. They argued for a connection between greater ventral striatum reactivity and functional dopaminergic variants that result in decreased postsynaptic dopamine receptor density. However, there is no clear-cut evidence concerning the functionality of these dopaminergic polymorphisms. The reduced DRD4 expression of the 7-repeat allele [27] has not been replicated. The *in vitro *reporter gene experiment of the DRD2 -141C Del allele showed lower expression [55], whereas a SPECT study showed higher striatal dopamine receptor density for the -141C Del variant [21]. Finally, several studies indicated that the 40 bp VNTR in the DAT1 gene 3' untranslated region affects gene expression, but the results are controversial concerning both the gene expression analyses [56,57] and the SPECT studies [58-60].

A more recent report showed an association between a reward-related impulsivity endophenotype in response to a psychological stressor and the DRD2 C957T polymorphism among healthy adults [61]. Although the findings of the *in vitro *and *in vivo *characterization of this SNP were controversial [62,63], a follow-up PET study indicated that the increased binding potential of the T-allele was more important than the slightly increased DRD2 density, making the DRD2 availability of the T-allele higher compared to the C-allele [64]. To date, only the reduced DRD2 density of the TaqI A1-allele carriers has been convincingly replicated [19-22]. However, the DRD2/ANKK1 TaqIA polymorphism is probably not directly involved in DRD2 gene expression. The TaqIA SNP (rs1800497) is in linkage with TaqIB SNP (rs1079597) and with C957T SNP (rs6277) within the DRD2 gene, and these SNPs have also been associated with altered DRD2 density [21,64]. In addition, the TaqIA SNP is in linkage with a couple of non-synonymous SNPs of the ANKK1 gene [65]. Interestingly, a neighboring non-synonymous SNP (rs273849, Arg490His) of the ANKK1 gene has been recently shown to alter NF-κB function, which in turn may affect DRD2 expression [66]. Therefore, the DRD2 TaqIA polymorphism might still serve as a genetic marker in psychogenetic studies, as indicated by meta-analyses of alcohol and substance misuse [67,68].

## Conclusions

Significant association was found between the DRD4 -616 CC genotype and BPD traits among US at-risk young adults, which was replicated in a Hungarian psychiatric patient sample. Association of the DRD2 TaqI B1-allele and A1-allele was also observed among the US young adults, however, this association was not present in the Hungarian inpatient psychiatric sample. The association between the indicated dopaminergic SNPs and BPD traits among young adults of low socioeconomic status remained significant after controlling for variance related to severity of abuse in the logistic regression equation. The role of the COMT or DAT1 polymorphisms in BPD symptom development among at-risk young adults or psychiatric patients was not supported by the present association analysis. Our results highlight the possible involvement of dopamine receptor variants in the development of BPD traits and call for further investigation of the dopamine system contribution in BPD.

## Competing interests

The authors declare that they have no competing interests.

## Authors' contributions

ZN conducted the genetic studies, participated in the study design, and drafted the manuscript. KLR and GF participated in the study design and acquisition of data in the US and Hungarian site, respectively. AS and EB participated in the analyses of the Hungarian and the US study, respectively. KLR, AS and MSS helped in revising the manuscript. MSS conceived of both genetic studies, and participated in design and coordination. All authors read and approved the final manuscript.

## Supplementary Material

Additional file 1 **Genotype and allele frequencies of dopaminergic polymorphisms in the US and Hungarian groups**. Genotype frequencies are shown in the upper part, allele frequencies are shown in the lower part of each dopaminergic polymorphism, namely the COMT Val158Met, the DAT1 40 bp VNTR, the DRD2 TaqIB, TaqID, TaqIA SNPs, and the DRD4 120 bp duplication, -616 C/G, -521 C/T, 48 bp VNTR. Click here for file
