# Supplementary material for: Association between dopaminergic polymorphisms and borderline personality traits among at-risk young adults and psychiatric inpatients
Source: Behav Brain Funct. 2010 Jan 12;6:4. doi: 10.1186/1744-9081-6-4 (PMC2823641; doi:10.1186/1744-9081-6-4)
Supplement: Additional file 1 — Genotype and allele frequencies of dopaminergic polymorphisms in the US and Hungarian groups. Genotype frequencies are shown in the upper part, allele frequencies are shown in the lower part of each dopaminergic polymorphism, namely the COMT Val158Met, the DAT1 40 bp VNTR, the DRD2 TaqIB, TaqID, TaqIA SNPs, and the DRD4 120 bp duplication, -616 C/G, -521 C/T, 48 bp VNTR. [file 1744-9081-6-4-S1.DOC]

Genotype and allele frequencies of dopaminergic polymorphisms in the US and Hungarian groups

|  |  | **US total**  **(N = 99)*** | | **US White (N = 66)*** | | **Hu patients (N = 136)** | | **Hu controls (N = 178)** | |
| --- | --- | --- | --- | --- | --- | --- | --- | --- | --- |
|  |  | **N** | **(%)** | **N** | **(%)** | **N** | **(%)** | **N** | **(%)** |
|  | **Met/Met** | 16 | (16.2) | 14 | (21.2) | 34 | (25.0) | 46 | (25.8) |
| **COMT** | **Met/Val** | 45 | (45.5) | 31 | (47.0) | 70 | (51.5) | 87 | (48.9) |
|  | **Val/Val** | 38 | (38.4) | 21 | (31.8) | 32 | (23.5) | 45 | (25.3) |
|  | **Met (A)** |  | (38.9) |  | (44.7) |  | (50.7) |  | (50.3) |
|  | **Val (G)** |  | (61.1) |  | (55.3) |  | (49.3) |  | (49.7) |
|  | **3/9** | 1 | (1.0) | - |  | - |  | - |  |
|  | **8/9** | 2 | (2.0) | - |  | - |  | - |  |
| **DAT1** | **9/9** | 10 | (10.2) | 7 | (10.6) | 11 | (8.1) | 23 | (12.9) |
| **40 bp** | **3/10** | 2 | (2.0) | - |  | - |  | - |  |
| **VNTR** | **6/10** | 1 | (1.0) | 1 | (1.5) | - |  | - |  |
|  | **9/10** | 29 | (29.6) | 20 | (30.3) | 48 | (35.3) | 62 | (34.8) |
|  | **10/10** | 51 | (52.0) | 35 | (53.0) | 75 | (55.1) | 91 | (51.1) |
|  | **10/11** | 2 | (2.0) | 2 | (3.0) | 1 | (0.7) | 2 | (1.1) |
|  | **10/12** | - |  | - |  | 1 | (0.7) | - |  |
|  | **3** |  | (1.5) |  | - |  | - |  | - |
|  | **6** |  | (0.5) |  | (0.8) |  | - |  | - |
|  | **8** |  | (1.0) |  | - |  | - |  | - |
|  | **9** |  | (26.5) |  | (26.2) |  | (25.7) |  | (30.3) |
|  | **10** |  | (69.4) |  | (71.5) |  | (73.5) |  | (69.1) |
|  | **11** |  | (1.0) |  | (1.5) |  | (0.4) |  | (0.6) |
|  | **12** |  | - |  | - |  | (0.4) |  | - |
|  | **B1/B1** | 1 | (1.0) | 1 | (1.6) | 3 | (2.2) | 3 | (1.7) |
| **DRD2** | **B1/B2** | 26 | (26.8) | 22 | (34.4) | 37 | (27.2) | 41 | (23.0) |
| **TaqIB** | **B2/B2** | 70 | (72.2) | 41 | (64.1) | 96 | (70.6) | 134 | (75.3) |
|  | **B1 (A)** |  | (14.4) |  | (18.8) |  | (15.8) |  | (13.2) |
|  | **B2 (G)** |  | (85.6) |  | (81.3) |  | (84.2) |  | (86.8) |
|  | **C/C** | 27 | (27.8) | 10 | (15.6) | 27 | (19.9) | 30 | (16.9) |
|  | **C/T** | 44 | (45.4) | 33 | (51.6) | 59 | (43.4) | 86 | (48.3) |
| **TaqID** | **T/T** | 26 | (26.8) | 21 | (32.8) | 50 | (36.8) | 62 | (34.8) |
|  | **C** |  | (50.5) |  | (41.4) |  | (41.5) |  | (41.0) |
|  | **T** |  | (49.5) |  | (58.6) |  | (58.5) |  | (59.0) |
|  | **A1/A1** | 6 | (6.1) | 4 | (6.1) | 4 | (2.9) | 3 | (1.7) |
|  | **A1/A2** | 38 | (38.4) | 22 | (33.3) | 41 | (30.1) | 53 | (29.8) |
| **TaqIA** | **A2/A2** | 55 | (55.6) | 40 | (60.6) | 91 | (66.9) | 122 | (68.5) |
|  | **A1 (T)** |  | (25.3) |  | (22.7) |  | (18.0) |  | (16.6) |
|  | **A2 (C)** |  | (74.7) |  | (77.3) |  | (82.0) |  | (83.4) |
|  | **1/1** | 5 | (5.1) | 2 | (3.0) | 4 | (2.9) | 1 | (0.6) |
| **DRD4** | **1/2** | 35 | (35.4) | 24 | (36.4) | 40 | (29.4) | 47 | (26.4) |
| **120 bp** | **2/2** | 59 | (59.6) | 40 | (60.6) | 92 | (67.6) | 130 | (73.0) |
|  | **1** |  | (22.7) |  | (21.2) |  | (17.6) |  | (13.8) |
|  | **2** |  | (77.3) |  | (78.8) |  | (82.4) |  | (86.2) |
| **–616** | **C/C** | 23 | (23.2) | 20 | (30.3) | 44 | (32.4) | 54 | (30.3) |
|  | **C/G** | 50 | (50.5) | 30 | (45.5) | 56 | (41.2) | 90 | (50.6) |
|  | **G/G** | 26 | (26.3) | 16 | (24.2) | 36 | (26.5) | 34 | (19.1) |
|  | **C** |  | (48.5) |  | (53.0) |  | (52.9) |  | (55.6) |
|  | **G** |  | (51.5) |  | (47.0) |  | (47.1) |  | (44.4) |
| **–521** | **C/C** | 13 | (13.1) | 7 | (10.6) | 34 | (25.0) | 38 | (21.3) |
|  | **C/T** | 52 | (52.5) | 35 | (53.0) | 65 | (47.8) | 93 | (52.2) |
|  | **T/T** | 34 | (34.3) | 24 | (36.4) | 37 | (27.2) | 47 | (26.4) |
|  | **C** |  | (39.4) |  | (37.1) |  | (48.9) |  | (47.5) |
|  | **T** |  | (60.6) |  | (62.9) |  | (51.1) |  | (52.5) |
| **48 bp** | **2/2** | 1 | (1.0) | 1 | (1.5) | 1 | (0.7) | - |  |
| **VNTR** | **2/3** | 2 | (2.0) | 2 | (3.0) | 1 | (0.7) | 1 | (0.6) |
|  | **2/4** | 9 | (9.1) | 5 | (7.6) | 14 | (10.3) | 23 | (12.9) |
|  | **2/7** | 1 | (1.0) | - |  | 2 | (1.5) | 9 | (5.1) |
|  | **2/8** | - |  | - |  | - |  | 1 | (0.6) |
|  | **3/3** | - |  | - |  | 2 | (1.5) | - |  |
|  | **3/4** | 9 | (9.1) | 9 | (13.6) | 5 | (3.7) | 4 | (2.2) |
|  | **3/7** | - |  | - |  | 3 | (2.2) | 5 | (2.8) |
|  | **4/4** | 46 | (46.5) | 30 | (45.5) | 71 | (52.2) | 83 | (46.6) |
|  | **4/5** | 1 | (1.0) | - |  | 1 | (0.7) | 2 | (1.1) |
|  | **4/6** | 1 | (1.0) | 1 | (1.5) | - |  | 1 | (0.6) |
|  | **4/7** | 24 | (24.2) | 15 | (22.7) | 29 | (21.3) | 41 | (23.0) |
|  | **4/8** | - |  | - |  | 2 | (1.5) | - |  |
|  | **4/9** | - |  | - |  | 1 | (0.7) | - |  |
|  | **5/7** | - |  | - |  | - |  | 2 | (1.1) |
|  | **5/8** | 1 | (1.0) | - |  | - |  | - |  |
|  | **7/7** | 4 | (4.0) | 3 | (4.5) | 3 | (2.2) | 4 | (2.2) |
|  | **7/8** | - |  | - |  | 1 | (0.7) | 2 | (1.1) |
|  | **2** |  | (7.1) |  | (6.8) |  | (7.0) |  | (9.6) |
|  | **3** |  | (5.6) |  | (8.3) |  | (4.8) |  | (2.8) |
|  | **4** |  | (68.7) |  | (68.2) |  | (71.3) |  | (66.6) |
|  | **5** |  | (1.0) |  | - |  | (0.4) |  | (1.1) |
|  | **6** |  | (0.5) |  | (0.8) |  | - |  | (0.3) |
|  | **7** |  | (16.7) |  | (15.9) |  | (15.1) |  | (18.8) |
|  | **8** |  | (0.5) |  | - |  | (1.1) |  | (0.8) |
|  | **9** |  | - |  | - |  | (0.4) |  | - |

* In the US group, one DNA sample did not give genotype result at the DAT1 VNTR (total sample size therefore was 98), whereas two DNA samples did not give genotype results at the DRD2 TaqIB and TaqID polymorphisms (sample size was 97 for the total sample, and 64 for the Caucasian subgroup).
